# Association between Chronic Prostatitis/Chronic Pelvic Pain Syndrome and Anxiety Disorder: A Population-Based Study

**DOI:** 10.1371/journal.pone.0064630

**Published:** 2013-05-15

**Authors:** Shiu-Dong Chung, Herng-Ching Lin

**Affiliations:** 1 Division of Urology, Department of Surgery, Far Eastern Memorial Hospital, New Taipei City, Taiwan; 2 Sleep Research Center, Taipei Medical University Hospital, Taipei, Taiwan; 3 School of Health Care Administration, Taipei Medical University, Taipei, Taiwan; Northwestern University, United States of America

## Abstract

**Background:**

this case-control study utilized a population-based dataset to examine the association of chronic prostatitis/chronic pelvic pain syndrome (CP/CPPS) with prior anxiety disorder (AD) by comparing the risk of prior AD between subjects with CP/CPPS and matched controls in Taiwan.

**Methods:**

We study used data sourced from the Taiwan Longitudinal Health Insurance Database. The cases comprised 8,088 subjects with CP/CPPS and 24,264 randomly matched subjects as controls. We used a conditional logistic regression to calculate the odds ratio (OR) for having been previously diagnosed with AD between subjects with and without CP/CPPS.

**Results:**

Of the 24,264 sampled subjects, 2309 (7.1%) had received an AD diagnosis before the index date; AD was found in 930 (11.5%) cases and 1379 (5.7%) controls (*p*<0.001). The conditional logistic regression analysis revealed that compared to controls, the OR for prior AD among cases was 2.10 (95% CI = 1.92∼2.29, *p*<0.001) after adjusting for diabetes, hypertension, hyperlipidemia, and sexually transmitted diseases. Our results show that CP/CPPS was consistently and significantly associated with prior AD in all age groups (18∼39, 40∼59, and >59 years). In particular, subjects aged 40∼59 years had the highest adjusted OR (of 2.53) for prior AD among cases compared to controls.

**Conclusions:**

We concluded that CP/CPPS is associated with previously diagnosed AD. Urologists should be alert for the association between CP/CPPS and AD in subjects suffering from AD.

## Introduction

Chronic prostatitis/chronic pelvic pain syndrome (CP/CPPS) is characterized by pelvic or perineal chronic pain in the tip of the penis, suprapubic region, or scrotum lasting longer than 3 months [1]. The etiology, pathogenesis, and risk factors for CP/CPPS are still unknown. Many researchers have proposed different etiologies and mechanisms for the pathogenesis of CP/CPPS including immunological, neurological, and endocrine factors [2]–[4]. In addition to the interplay among the immune, endocrine, and nervous systems, psychological factors may also play important roles in producing CP/CPPS symptoms [5].

Of all types of psychological disorders, anxiety disorder (AD) is one of the most highly prevalent in the general population with a worldwide lifetime prevalence ranging 9.2%∼28.7% [6], [7]. AD commonly occurs along with other mental or physical illnesses; moreover, there is increasing evidence that anxiety is associated with high rates of medically unexplained symptoms [8]–[10]. In particular, an accumulating body of research has suggested an association between anxiety/panic disorder/symptoms and CP/CPPS [1], [11]–[15]. However, most studies were based on patient self-reported surveys rather than on confirmed physician diagnoses. Only one study by Clemens et al. used clinical electronic medical records to assess the relationship between medical comorbidities and a prostatitis diagnosis [12]. Therefore, those studies may have all been subject to recall bias. In addition, no such studies explored the causal relationship between CP/CPPS and AD because of their cross-sectional design. This prevents clinicians and researchers from understanding possible etiologies of CP/CPPS from a psychological perspective.

To fill in this gap in the literature, this case-control study utilized a population-based dataset to examine the association of CP/CPPS with prior AD by comparing the risk of prior AD between subjects with CP/CPPS and matched controls in Taiwan.

## Methods

### Database

Sampled subjects analyzed in this study were retrieved from the Longitudinal Health Insurance Database 2000 (LHID2000). The LHID2000, which is derived from medical claims records of the Taiwan National Health Insurance (NHI) program, includes all medical claims and registration files for 1,000,000 enrollees under the Taiwan NHI program randomly sampled from all enrollees listed in the 2000 Registry of Beneficiaries (*n* = 23.72 million). The Taiwan National Health Research Institute and researchers have demonstrated the high validity of the data from the NHI program [16], [17]. Furthermore, hundreds of papers employing the LHID2000 have been published in internationally peer-reviewed journals [18].

This study was exempt from full review by the Institutional Review Board of Taipei Medical University because the LHID2000 consists of de-identified secondary data released to the public for research purposes.

### Selection of Cases and Controls

As to the selection of cases, we first identified 9312 subjects who had received a first-time diagnosis of CP/CPPS (ICD-9CM code 601.1) during an ambulatory care visit (including outpatient departments of hospitals and clinics) from January 1, 2001 to December 31, 2011. We excluded those subjects aged less than 18 years (*n* = 107) in order to limit the study sample to the adult population. We further assigned their first ambulatory care visit for the treatment of CP/CPPS as the index date. In Taiwan, CP/CPPS is diagnosed symptomatically, and is characterized by a 3-month history of genitourinary pain and an absence of other lower urinary tract pathologies. However, there is no gold standard for diagnosing CP/CPPS to date, so this study only included those patients who had received two or more CP/CPPS diagnoses with at least one being made by a urologist in order to increase the diagnostic validity of CP/CPPS (*n* = 9010). In addition, we did not include a CP/CPPS case if a subject received a diagnosis of prostate cancer, inguinal hernia, interstitial cystitis, urethritis, or benign enlargement of the prostate within 1 year prior to the index date (*n* = 802) in order to eliminate the possibility of other diseases being confused with CP/CPPS. Finally, we excluded those subjects who had a history of major psychosis (except AD) or a substance-related disorder (ICD-9-CM codes 290∼299 or 303∼305) prior to the index date (*n* = 120). As a result, 8088 subjects with CP/CPPS were included as cases.

We also retrieved controls from the LHID2000. We randomly selected 24,264 controls to match the cases (3 controls per case) in terms of age group (18∼29, 30∼34, 35∼39, 40∼44, 45∼49, 50∼54, 55∼59, 60∼64, 65∼69, and >69 years), geographic region of residence, urbanization level, and index year. We also determined that none of the selected controls had ever received a diagnosis of CP/CPPS, major psychosis (except AD), or substance-related disorder since inauguration of the NHI program. For the controls, we assigned their first utilization of medical care occurring in the index year as their index date.

### Exposure Assessment

This study included cases with AD (panic disorder, agoraphobia, specific phobias, social phobias, obsessive-compulsive disorder, posttraumatic stress disorder, acute stress disorder, and generalized anxiety disorders) based on ICD-9-CM codes 300.01, 300.02, 300.2, 300.20∼300.29, 300.3, 308, and 309.81∼309.83. In addition, we only selected cases who had received an AD diagnosis within 3 years prior to the index date. We also ensured that all AD cases included in this study were diagnosed by a certified psychiatrist.

### Statistical Analysis

We used the SAS system (SAS System for Windows, vers. 8.2, SAS Institute, Cary, NC) to conduct all statistical analyses. We used a conditional logistic regression (conditioned on the age group, geographic region, urbanization level, and index year) to calculate the odds ratio (OR) and 95% confidence interval (CI) for having been previously diagnosed with AD between subjects with and those without CP/CPPS. We also took medical comorbidities including diabetes, hypertension, obesity, hyperlipidemia, sexually transmitted diseases, and a history of a vasectomy into consideration in the regression model. The conventional *p* ≤ 0.05 was used to assess the statistical significance.

## Results

Table 1 shows the demographic characteristics and medical comorbidities according to the presence of CP/CPPS. After matching for age group, geographic region, urbanization level, and index year, cases had a higher prevalence than controls of diabetes (16.7% vs. 14.9%, *p* = 0.002), hypertension (33.3% vs. 29.9%, *p*<0.001), hyperlipidemia (26.3% vs. 21.3%, *p*<0.001), and sexually transmitted diseases (1.9% vs. 0.6%, *p*<0.001). However, there was no significant difference in the prevalence of obesity or a history of a vasectomy between cases and controls.

**Table 1:** Demographic characteristics of subjects with chronic prostatitis/chronic pelvic pain syndrome and controls in Taiwan (N = 32,352).

Variable	Subjects with chronic prostatitis/chronic pelvic pain syndrome(*n* = 8088)		Controls(*n* = 24,264)		*p* value	
	Total no.	Percent	Total no.	Percent		
Age (years)					1.000	
18∼29	802	9.9	2406	9.9		
30∼34	601	7.4	1803	7.4		
35∼39	744	9.2	2232	9.2		
40∼44	870	10.8	2610	10.8		
45∼49	962	11.9	2886	11.9		
50∼54	899	11.1	2697	11.1		
55∼59	890	11.0	2670	11.0		
60∼64	801	9.9	2403	9.9		
65∼69	788	9.8	2364	9.8		
>70	731	9.0	2193	9.0		
Geographic region					1.000	
Northern	4528	56.0	13,584	56.0		
Central	1697	21.0	5091	21.0		
Eastern	1732	21.4	5196	21.4		
Southern	131	1.6	393	1.6		
Urbanization level					1.000	
1 (most urbanized)	2968	36.7	8904	36.7		
2	2169	26.8	6507	26.8		
3	1137	14.1	3411	14.1		
4	950	11.7	2850	11.7		
5 (least urbanized)	864	10.7	2592	10.7		
**Variable**	**Subjects with chronic prostatitis/chronic pelvic pain syndrome** **(** ***n*** ** = 8088)**		**Controls** **(** ***n*** ** = 24,264)**		***p*** ** value**	
	**Total no.**	**Percent**	**Total no.**	**Percent**		
Monthly income					<0.001	
NT$0∼15,840	2,584	31.9	7,990	32.9		
NT$15,841∼25,000	2,457	30.4	8,238	33.9		
≥NT$25,001	3,047	37.7	8,036	33.2		
Hyperlipidemia	2,123	26.3	5,178	21.3	<0.001	
Diabetes	1,349	16.7	3,623	14.9	0.002	
Hypertension	2,689	33.3	7,262	29.9	<0.001	
Obesity	55	0.6	160	0.7	0.843	
Sexually transmitted diseases	160	1.9	139	0.6	<0.001	
A history of a vasectomy	6	0.1	9	0.1	0.179	

In 2011, the average exchange rate was US$1.00≈New Taiwan Dollar (NT$).

Table 2 presents the prevalence of prior AD between cases and controls. Of the 24,264 sampled subjects, 2309 (7.1%) had received an AD diagnosis before the index date. AD was found in 930 (11.5%) cases and 1379 (5.7%) controls (*p*<0.001). Furthermore, the conditional logistic regression analysis revealed that compared to the controls, the OR for prior AD among cases was 2.10 (95% CI = 1.92∼2.29, *p*<0.001) after adjusting for diabetes, hypertension, hyperlipidemia, and sexually transmitted diseases.

**Table 2:** Odds ratios (ORs) for previous anxiety disorder among the sample subjects (N = 24,264).

Variable	Total sample		Controls		Subjects with chronic prostatitis/chronic pelvic pain syndrome	
	No.	Percent	No.	Percent	No.	Percent
Presence of prior anxiety disorder	2309	7.1	1379	5.7	930	11.5
Crude OR (95% confidence interval)	–		1.00		2.16*** (1.98∼2.35)	
Adjusted OR^a^ (95% confidence interval)	–		1.00		2.10*** (1.92∼2.29)	

*** *p*<0.001. The odds ratio was calculated using a conditional logistic regression (conditioned on age group, urbanization level, and index year) which was performed to adjust for hypertension, diabetes, hyperlipidemia, and sexually transmitted diseases.

Table 3 shows the OR for prior AD stratified by age group. It shows that CP/CPPS was consistently and significantly associated with prior AD in all age groups. In particular, subjects aged 40∼59 had the highest adjusted OR for prior AD among cases compared to the controls (OR = 2.53; 95% CI = 2.21∼2.89; *p*<0.001).

**Table 3:** Odds ratios (ORs) for previous anxiety disorder among subjects with chronic prostatitis/chronic pelvic pain syndrome and comparison group, by age group.

Presence of previous anxiety disorder	Age group (years)					
	18∼39		40∼59		>59	
	Subjects with chronic prostatitis/chronic pelvic pain syndrome *n*, Percent	Controls*n*, Percent	Subjects with chronic prostatitis/chronic pelvic pain syndrome*n*, Percent	Controls*n*, Percent	Subjects with chronic prostatitis/chronic pelvic pain syndrome*n*, Percent	Controls*n*, Percent
Yes	196 (9.1)	277 (4.3)	424 (11.7)	539 (5.0)	310 (13.4)	563 (8.1)
Crude OR (95% CI)	2.25*** (1.86∼2.72)	1.00	2.55*** (2.22∼2.91)	1.00	1.76*** (1.52∼2.04)	1.00
Adjusted OR^a^ (95% CI)	2.22*** (1.83∼2.70)	1.00	2.53*** (2.21∼2.89)	1.00	1.63*** (1.40∼1.89)	1.00

*** *p*<0.001. CI, confidence interval. The odds ratio was calculated using a conditional logistic regression (conditioned on urbanization level and index year) which was performed to adjust for hypertension, diabetes, hyperlipidemia, and sexually transmitted diseases.

Table 4 shows results of the sensitivity analysis in order to reduce the potential bias caused by the long AD diagnostic latency period. After excluding subjects who were diagnosed with AD within 1 and 2 years prior to the index date, the respective adjusted ORs were 1.89 and 2.19. This provides further evidence supporting the association between CP/CPPS and prior AD.

**Table 4:** Sensitivity analysis.

Presence of prior anxiety disorder	Excluding subjects who received an anxiety disorder diagnosis within 1 year prior to the index date		Excluding subjects who received an anxiety disorder diagnosis within 2 years prior to the index date	
	Subjects with chronic prostatitis/chronic pelvic pain syndrome*n* = 7820	Controls*n* = 23,979	Subjects with chronic prostatitis/chronic pelvic pain syndrome*n* = 7686	Controls*n* = 23,631
	*n* (Percent)			
Yes	662 (8.5)	1,094 (4.6)	528 (6.9)	746 (3.2)
Crude OR (95% CI)	1.94*** (1.75∼2.14)		2.26*** (2.02∼2.54)	
Adjusted OR^a^ (95% CI)	1.89*** (1.71∼2.09)		2.19*** (1.95∼2.46)	

*** *p*<0.001. CI, confidence interval; OR, odds ratio. The odds ratio was calculated using a conditional logistic regression (conditioned on age group, urbanization level, and index year) which was performed to adjust for hypertension, diabetes, hyperlipidemia, and sexually transmitted diseases.

## Discussion

The association between psychological disorders and CP/CPPS has recently garnered much attention. In this case-control study, we found that subjects with CP/CPPS had a significantly higher prevalence of prior AD than the matched controls (11.5% vs. 5.7%). We also found that the OR for prior AD among subjects with CP/CPPS was 2.10 compared to controls, after taking subject sociodemographic characteristics, diabetes, hypertension, hyperlipidemia, and sexually transmitted diseases into consideration. This finding was consistent with prior studies, all of which observed a higher prevalence of anxiety/panic disorder/symptoms in men with CP/CPPS than the controls [1], [11]–[15]. We further excluded subjects who had been diagnosed with AD within 1 and 2 years prior to the index date, and the association between CP/CPPS and prior AD still remained. This strong association might imply important clues to the psychological pathogenesis and pathophysiology of CP/CPPS.

However, the mechanisms underlying the association of AD with CP/CPPS remain unclear. It was suggested that stress accompanied by anxiety is a potent factor in the development, prolongation, and perpetuation of CP/CPPS symptoms [19], [20]. One experimental study also confirmed that chronic stress in rats can specifically induce histological inflammation of the prostate [21]. Chronic activation of the physiologic stress response augments release of proinflammatory cytokines and prostaglandins that may contribute to CP/CPPS syndromes [22]. There appear to be some measurable effects of stress changes on cytokine levels in patients with CP/CPPS [3]. In addition, stress and anxiety can influence HPA axis responses. Studies showed that HPA dysregulation may lead to abnormalities of inflammatory responses, resulting in chronic inflammatory and pain conditions such as interstitial cystitis or CP/CPPS [5], [23].

CP/CPPS and AD may also originate from a shared genetic susceptibility. Among all categories of AD, panic disorder was mentioned as being highly associated with CP/CPPS. Some prior studies documented that patients with panic disorder were more likely to have symptoms of bladder pain syndrome/interstitial cystitis (BPS/IC) [24], [25], which may represent the same underlying condition as CP/CPPS [26]. The common genetic susceptibility possibly shared by BPS/IC and AD might be linked to the Barrington nucleus, which provides an anatomical substrate for co-regulation of pelvic visceral symptoms and mental activity in the prosencephalon [27]. AD and panic disorder are often comorbid with one another due to the similarity of their causes, meaning that those who suffer from one often suffer from the other, or will later in life. Therefore, despite lacking genetic data regarding the association between CP/CPPS and AD, a common genetic vulnerability might be one possible explanation.

The strength of our study lies in its large population-based database. Its use allowed us to avoid many of the problems, such as selection biases, inherent in studies utilizing data taken from voluntary registries or hospital-referred study patients. Furthermore, unlike prior studies which used questionnaires, use of this population-based dataset could successfully avoid the effect of recall bias.

Nevertheless, some limitations of this study need to be addressed. First, AD and CP/CPPS diagnoses relied on administrative claims data reported by physicians and hospitals, and may be less accurate than diagnoses made according to standardized criteria. To avoid mistaken diagnoses, we only selected subjects who had been diagnosed with CP/CPPS at least once by a urologist. However, although chronic bacterial prostatitis was found to only account for 4.2% of men with prostatitis syndrome [28], the dataset used in this study did not allow us to differentiate chronic bacterial prostatitis from CP/CPPS.

Second, the dataset did not contain information on some variables such as stressful psychosocial life events, dietary habits, physical activities, smoking status, psychotropic medications, and non-pharmacological treatments, which may have contributed to the link.

Finally, this investigation utilized a case-control study design. Although we performed a sensitivity analysis in order to reduce the potential bias caused by the long AD diagnostic latency period, the association between CP/CPPS and AD only weakly suggested temporality and could not be used to establish causality.

Despite the above limitations, our study demonstrated an association between CP/CPPS and previously diagnosed AD. Urologists should be alert to the association between CP/CPPS and AD in subjects suffering from AD. We suggest that prospective studies be conducted to better understand the causal relationship between AD and CP/CPPS. In addition, further studies are needed to clarify the mechanism between AD and CP/CPPS.
